# The Role of Wnt Pathway in the Pathogenesis of OA and Its Potential Therapeutic Implications in the Field of Regenerative Medicine

**DOI:** 10.1155/2018/7402947

**Published:** 2018-10-15

**Authors:** Maria De Santis, Berardo Di Matteo, Emanuele Chisari, Gilberto Cincinelli, Peter Angele, Christian Lattermann, Giuseppe Filardo, Nicolò Danilo Vitale, Carlo Selmi, Elizaveta Kon

**Affiliations:** ^1^Division of Rheumatology and Clinical Immunology, Humanitas Research Hospital, Rozzano, Milan, Italy; ^2^Humanitas University Department of Biomedical Sciences, Via Manzoni 113, 20089 Rozzano, Milan, Italy; ^3^Humanitas Clinical and Research Center, Via Manzoni 56, 20089 Rozzano, Milan, Italy; ^4^Department of General Surgery and Medical Surgery Specialties, Section of Orthopaedics and Traumatology, University Hospital Policlinico-Vittorio Emanuele, University of Catania, Catania, Italy; ^5^Department of Trauma Surgery, University Medical Center Regensburg & Sporthopaedicum Straubing, Regensburg, Germany; ^6^Brigham and Women's Hospital, Harvard Medical School, Chestnut Hill, MA, USA; ^7^Applied and Translational Research Center, IRCCS Istituto Ortopedico Rizzoli, Bologna, Italy; ^8^BIOMETRA Department, University of Milan, Milan, Italy

## Abstract

**Introduction:**

Osteoarthritis (OA) is a degenerative joint disease characterized by articular cartilage degradation, subchondral damage, and bone remodelling, affecting most commonly weight-bearing joints, such as the knee and hip. The loss of cartilage leads to joint space narrowing, pain, and loss of function which could ultimately require total joint replacement. The Wnt/*β* catenin pathway is involved in the pathophysiology of OA and has been proposed as a therapeutic target. Endogenous and pharmacological inhibitors of this pathway were recently investigated within innovative therapies including the use of platelet-rich plasma (PRP) and mesenchymal stem cells (MSCs).

**Methods:**

A review of the literature was performed on the PubMed database based on the following inclusion criteria: article written in English language in the last 20 years and dealing with (1) the role of Wnt-*β* catenin pathway in the pathogenesis of osteoarthritis and (2) pharmacologic or biologic strategies modulating the Wnt-*β* catenin pathway in the OA setting.

**Results:**

Evidences support that Wnt signalling pathway is likely linked to OA progression and severity. Its inhibition through natural antagonists and new synthetic or biological drugs shares the potential to improve the clinical condition of the patients by affecting the pathological activity of Wnt/*β*-catenin signalling.

**Conclusions:**

While further research is needed to better understand the mechanisms regulating the molecular interaction between OA regenerative therapies and Wnt, it seems that biologic therapies for OA exert modulation on Wnt/*β* catenin pathway that might be relevant in achieving the beneficial clinical effect of those therapeutic strategies.

## 1. Introduction

Osteoarthritis (OA) is a degenerative joint disease characterized by articular cartilage degradation, subchondral damage, and bone remodelling, affecting most commonly weight-bearing joints such as the knee and hip. Many treatment options are currently available for OA, ranging from conservative to surgical measures and regenerative medicine approaches. Despite wide research efforts on OA, there is a huge unmet need in effective therapies that ultimately change the natural history of the disease. Recently introduced autologous treatments, such as platelet-rich plasma (PRP) and mesenchymal stem cells (MSC), have been largely investigated in orthopaedic surgery and proposed as OA treatments. The rationale for the use of these biologic products is based on their capability of modulating the joint environment by releasing a series of growth factors and immune-modulatory molecules that could play a beneficial role in reducing the local inflammation and promoting cartilage and synovium anabolism [1]. From a pathogenetic standpoint, cartilage homeostasis and bone remodelling are regulated by a complex network of metabolic pathways and, among these, the Wnt/*β*-catenin pathway is activated in OA and is emerging as a regulator of tissue repair and fibrosis [2]. It has been hypothesized that PRP may protect chondrocytes activated by IL-1*β* via inhibiting Wnt/*β*-catenin signalling [3]. The aim of the present review is to shed light on the current concepts concerning the role of the Wnt/*β*-catenin pathway in the pathogenesis of OA, and understanding the potential therapeutic implications in the field of regenerative medicine.

## 2. Methods

A review of the literature was performed on the PubMed database by two independent authors (CE and VND). The research string used was the following:

“((Wnt OR catenin) OR (IL 1 AND Wnt) OR (dkk1 and Wnt)) AND (osteoarthritis treatment OR PRP OR platelet rich plasma OR (MSC AND osteoarthritis))”

The inclusion criteria were in vitro articles written in English language, published in the last 20 years, and dealing with (1) the role of the Wnt-*β* catenin pathway in the pathogenesis of osteoarthritis and (2) pharmacologic or biologic strategies modulating the Wnt-*β* catenin pathway in the OA setting.

A total of 168 articles were retrieved: first, the articles were screened by title and abstract and then the full texts of the selected articles were analyzed. Reference lists from the selected papers were also screened and, at the end of the selection process, 14 papers in total were included in the present review. Relevant data were then extracted and collected in a unique database with the consensus of the two aforementioned authors.

## 3. Results

Of the 14 articles included in this review [3–16] (Tables 1, 2, and 3), ten focused on the molecular mechanism in which OA, Wnt, and endogenous inhibitors are associated [5–14], two on PRP and its potential to modulate the Wnt pathway, and two on new potential pharmacological inhibitors [4, 16]. Most of the studies reported how the Wnt inhibition can be a potential new target for OA treatment and explored how this can improve the clinical outcome of patients. Early investigations of the Wnt/*β*-catenin pathway modulation with biologicals and regenerative therapies demonstrate promise. However, in the absence of any long-term clinical data in a substantial number of clinical subjects, further study is required to elucidate proof of concept, safety, and efficacy. What emerged from the present review of the literature is an increasing interest in therapeutic approaches to influence this signalling way, especially with PRP, but also with other biologics, and new experimental inhibitors.

### 3.1. Wnt Canonical and Noncanonical Signalling Pathways: A Comprehensive Synthesis

The Wnt family of proteins includes morphogens associated with embryonic formation, tissue repair, fibrosis, and homeostasis of the bone and joint tissues, among others [4, 17].

Wnt regulates multiple signalling cascades, including one *β*-catenin–dependent (canonical) and two *β*-catenin–independent pathways [18]. The Wnt/*β*-catenin canonical signalling pathway is initiated by the binding of the Wnt proteins to their 7-pass transmembrane Frizzled (Fz) receptors and results in the activation of the central player of the pathway, downstream *β*-catenin. To activate the canonical signalling pathway, Wnt ligands must bind to both Fz receptors and the coreceptor low-density lipoprotein receptor-related protein (LRP) 5/6. This binding further enables the release of *β*-catenin from its intracellular binding complex [composed of glycogen synthase kinase-3*β* (GSK3*β*), caseine kinase 1-*α* (CK1*α*), adenomatous polyposis coli (APC), and Axin2], which in the Wnt-off state phosphorylates and ubiquitinates *β*-catenin, leading to its degradation in proteasomes [19, 20]. Alternatively, in the Wnt-on state, or when a Wnt ligand is available, Fz receptors and LRP 5/6 are activated upon ligand binding. The receptor occupancy leads to the phosphorylation of LRP 5/6 by Gsk3*β* and Ck1*α* in its cytoplasmic region, followed by the recruitment of the dishevelled (DVL)1–3 andaxin [20], which inhibits the destruction of the complex and the stabilization of *β*-catenin in the cytoplasm. The activated *β*-catenin translocates into the nucleus where it binds the transcription factors of T cell factor (TCF)/lymphoid enhancer factor (LEF) transcription factors, converting them from repressors to activators causing the transcription of targeted genes [21, 22]. The two noncanonical pathways include a Ca^2+^ dependent (transmitted via calmodulin kinase II and protein kinase C) and a planar cell polarity pathway (transmitted via small GTPases) and both contribute to the regulation of cytoskeletal organisation, cell differentiation, and communication [22–24]. Members of the Wnt ligand family, currently including 19 members, activate either the canonical or the noncanonical pathway, according to the receptor they bind to as mentioned above [25]. Ultimately, both *β*-catenin dependent and independent cascades are demonstrated in the joint where the noncanonical pathways appear to counterregulate the canonical Wnt pathway [26].

The Wnt signalling pathways also undergo strict regulation by different inhibitors, including the family of secreted frizzled related proteins (SFRP) 1-4 and Wnt inhibitor factor (WIF) which act as a soluble scavenger of Wnt ligands [27]; other molecules, such as members of Dickkopf (DKK) family and sclerostin, bind to LRP 5/6 and interfere with the Wnt downstream activation cascade disabling their ability to interact with Wnt-Fz [17, 28], among other inhibitors [28].

The dysregulation of the Wnt canonical pathway is believed to be central to the pathogenesis of cancer, chronic inflammation, and degenerative diseases, and natural inhibitors are thus under investigation for therapeutic purpose. The mechanisms by which the Wnt pathway is influenced by inflammation remain unclear, but its involvement is well established in new bone formation through osteoblast differentiation and function, in the role of TNF*α* as a major driver of bone destruction in arthritis, and upregulating the Wnt antagonist Dkk-1 and inhibiting new bone formation. In particular, a prolonged mechanical stress can induce TNF-*α* but also IL-1 expression in chondrocytes [29], thus suggesting a possible role for both cytokines in OA.

### 3.2. Role of the Wnt Pathway in OA Development

The pathological processes involved in OA have been widely investigated during the last decades and can be summarized in articular cartilage degradation, subchondral bone remodeling, and synovitis regulated by a complex network of different molecular pathways, including the Wnt/*β*-catenin signaling network in the homeostasis of bone and cartilage [30–32]. WNTs, a large family of 19 secreted glycoproteins, bind their frizzled (FZD) receptors preventing *β*-catenin to be phosphorylated and disposed, leading to its accumulation and subsequent migration to the nucleus, where it activates the transcription of different target genes [33].

In OA, this pathway is enhanced in both human and animal models [6–9, 34]. There is an increase in the expression levels of different proteins involved in OA and facilitating cartilage matrix degradation, including MMP-13, ADAMTS-4, ADAMTS-5, and RUNX-2C [10]. One of the initial steps of OA physiopathology is the destruction of collagen type II (COL2) and this phenomenon is activated by the upregulation of WNT5A in rat model of OA-chondrocytes. Silencing WNT5A mRNA through delivering small interfering RNA by a lentiviral vector prevented degradation of COL2 [11]. The WNT/*β*-catenin pathway can also be activated by the induction of the histone methyltransferase enhancer of zeste homologue 2 (EZH2) [12]. EZH2 level was found significantly increased in chondrocytes of OA patients compared to healthy humans, along with high-level expression of MMP-13, ADAMTS-5, and COLX. Pharmacological inhibition of EZH2 silenced *β*-catenin signaling pathway and delayed OA progression in mice. Evidence from in vivo and in vitro studies indicates that synoviocytes, chondrocytes, and cells from other joint tissues produce cytokines in response to mechanical or oxidative stress or degradation products [35]. The in vitro mechanical loading data support the hypothesis that static compression stimulates the depletion of proteoglycans and damage to the collagen network and decreases the synthesis of cartilage matrix proteins, through the action of reactive oxygen species (ROS) and TNF-*α* [36], whereas dynamic cyclic compression increases matrix synthetic activity inhibiting IL-1-induced cartilage matrix degradation [37]. IL-1*β* is a key proinflammatory cytokine that drives OA progression by inducing the expression of cartilage degrading enzymes, such as matrix metalloproteinases (MMPs) [38] and nitric oxide (NO) expression involved in joint damage. NO is highly expressed by OA chondrocytes and cartilage and inhibits both the synthesis of proteoglycan and collagen, activates MMPs, mediates chondrocyte apoptosis, and promotes inflammatory responses, ultimately resulting in a major catabolic effect. A recent study on human chondrocytes reported that IL-1*β* decreases the expression of DKK1 and FRZB through the upregulation of nitric oxide synthase, and the activation of Wnt target gene transcription [39].

### 3.3. Genetic Variability of the Wnt Pathway

The key role of Wnt in joint homeostasis, OA development, and treatment is demonstrated by several studies, which provide evidence of the association between single nucleotide polymorphisms (SNPs) in different genes encoding for proteins of the pathway associated to disease. Variants of the gene of WNT inhibitor sFRP-3/FRZB were associated with OA, reducing its capacity to antagonizing *β*-catenin [40]. A SNP in the frizzled coreceptor LRP5 prevents the Wnt signaling antagonists SOST and Dkk-1 from binding, inducing spinal OA [41]. More recently, a SNP in WNT1 inducible signaling pathway protein 1 (WISP1), a Wnt-induced protein, has been related to spine OA [42]. Another important consideration about the Wnt/*β*-catenin pathway comes from a genome-wide analysis of gene expression in knee cartilage affected by OA [43]. Network analysis of RNA-sequencing identified the presence of two different major pathogenetic pathways: one with higher expression of chondrogenic genes and altered expression of chemokine signaling, inflammasome, and innate immune response and the other with a more osteogenic phenotype and altered WNT signaling and mechanoreceptors. This observation may well represent a new starting point for the stratification of patients with OA, based on the phenotype of the disease: i.e., inflammatory and load-related, thus allowing more specific and individualized targeted treatments.

### 3.4. Endogenous Inhibitors of Wnt

The Wnt pathway is controlled by endogenous antagonists and the expression levels of some of these inhibitors may decrease in parallel with OA progression, thus supporting a link between Wnt pathway and OA pathogenesis.

#### 3.4.1. Sclerostin

Bone tissue cells are actively involved in the development of OA by synthesizing molecules that regulate the joint homeostasis. In particular, osteocytes produce sclerostin, an endogenous Wnt/*β*-catenin inhibitor that plays an important role in maintaining cartilage integrity, as demonstrated in a study on a murine model of OA [5]. Healthy and OA chondrocytes were incubated with 250 ng/ml sclerostin for 48h and as a result decreased mRNA expression levels of *β*-catenin have been obtained in both groups. Treatment with sclerostin also inhibited the expression levels of downstream catabolic effector genes RUNX-2, MMP-13, ADAMTS-4, and ADAMTS-5 in healthy chondrocytes. Anabolic factor COL2A1 expression increased with sclerostin challenge and these effects have not been observed in OA chondrocytes compared with the control group, indicating that sclerostin may not have beneficial effects on OA chondrocytes. Chondrocytes were also incubated with 10 ng/l IL-1*α* for 48h, resulting in increased mRNA expression levels of *β*-catenin. Therefore IL-1*α* may control cartilage degradation via overactivation of the WNT/*β*-catenin pathway.

The protective role of sclerostin through the inhibition of Wnt was also suggested in another study [44]. Sclerostin-knockout mice with DMM had a high OA score, increased expression of aggrecanases and type X collagen, and cartilage damage with inhibition of both WNT canonical and noncanonical pathways.

#### 3.4.2. Wnt-Inhibitor Factor-1

The WNT-inhibitory factor 1 (WIF-1) is a potent extracellular WNT antagonist whose role has been investigated in many trials. Looking at its implications in articular degenerative pathology, it was found that patients with OA had significantly decreased WIF-1 levels compared to control, and WIF-1 levels also negatively correlated with the severity of the disease, as described by Gao et al. who analyzed specimens obtained from 40 patients with various stages of knee OA [13].

#### 3.4.3. Dickkopf

Dkk-1 is also a Wnt antagonist and is activated in osteocytes of the subchondral bone as well as in osteophytes and synovium after 4-6 weeks, as shown in a group of Topgal mice (transgenic mice that express Beta-galactosidase in the presence of the lymphoid enhancer binding factor 1/transcription factor 3 (LEF/TCF) and activated Beta-catenin) with OA induced by meniscectomy. The Dkk-1 expression is also elevated in chondrocytes of control mice but decreased greatly in knees of mice that underwent meniscectomy, starting from week 4 [45]. In a 2012 study, Dkk-1 overexpression by intra-articular injection significantly reduced progression of OA in mice induced with surgical destabilization of the medial meniscus. This was linked to Dkk-1 capacity to inhibit Wnt-mediated expression of catabolic factors, such as MMP-13, showing how Dkk-1 may exert a protective role in OA [14]. But data are conflicting. In 2010 Weng et al. demonstrated that elevated Dkk-1 levels were associated with a high Mankin score and high serum levels of cartilage degradation markers in murine models. Dkk-1 knockdown increased *β*-catenin but attenuated the expression of inflammatory factors (TLR-4, TLR-9, IL-1*β*, and TNF-*α*), the apoptosis regulator Bax, MMP-3, and RANK-L and these data suggest that Dkk-1 knockout produced less cartilage destruction and less subchondral damage in OA knee [46]. Dickkopf-3 (Dkk-3) is also a member of the Dkk family of WNT antagonists and elevated concentrations inhibited the loss of proteoglycan and collagen from cartilage in a model of OA obtained with IL-1*β* and oncostatin-M [47].

#### 3.4.4. Pharmacological Inhibitors of Wnt

Recently, new potential drugs which selectively inhibit the Wnt pathway have been investigated and in a phase 1 study, the inhibition of Wnt/*β*-catenin pathway in mice led to a less severe OA. The small-molecule “XAV-939”, a tankyrase inhibitor that promotes the phosphorylation of *β*-catenin leading to its destruction, was intra-articularly injected in a murine model of traumatic OA, obtained by a surgical destabilization of the medial meniscus and a reduced cartilage degradation and synovial inflammation was observed. XAV-939, along with the small-molecule inhibitor C113, was also tested in vitro on human synovial fibroblasts and OA-derived chondrocytes and led to an attenuated proliferation and type I collagen synthesis in synovial fibroblasts while in chondrocytes, although there was no affection in proliferation. In further detail, the Wnt inhibition led to an increased transcription of COL2A1 and PRG4, two genes which are downregulated in the cartilage affected by OA [4]. Another preclinical study evaluated the effectiveness of SM04690, a small-molecule Wnt pathway inhibitor, both in cultured human mesenchymal stem cells (hMSC) and in rodent OA model. The new potential drug induced hMSC differentiation into mature, functional chondrocytes and decreased cartilage catabolic marker levels compared to vehicle. A single SM04690 intra-articular (IA) injection was effective in a rodent OA model, with increased cartilage thickness, evidence for cartilage regeneration, and protection from cartilage catabolism [16].

#### 3.4.5. Biological Therapies and Wnt

PRP is an autologous blood derivate widely used for treating several musculoskeletal diseases and, despite limited evidence, its use has demonstrated promising results also in OA. The effects on OA progression and clinical outcome can be related to the growth factors contained in it. The effects of PRP on the WNT/*β*-catenin pathway were studied in New Zealand white rabbit chondrocytes. These chondrocytes were cultured, and a group was treated with PRP, whereas another group was treated by IL-1b: concentrations of cartilage degradation proteins CTX-II and COMP were lower in PRP group and less ultrastructural abnormalities and enhanced chondrocyte proliferation were also observed by electron microscopy [3]. Wang et al. reported that PRP inhibits bone degradation via downregulation of osteoclast-associated genes induced by RANKL. A gene expression profile chip analysis revealed the overexpression of WNT/*β*-catenin pathway genes and an important downregulation of Dkk1, which was demonstrated to enhance osteoclast genesis [48], thus proving conflicting data [15]. Similar to PRP, intra-articular MSC, both derived from the adipose tissue and bone marrow, have been used for the treatment of OA. The therapeutic mechanism involves Wnt pathway and MMP-13, TNF-*α*, and IL-1*β* expression [49], even though until now there is a lack of clinical efficacy evidence. As shown by Cassano et al, the growth factors concentrations of whole blood, PRP, and bone marrow concentrate differ significantly [50], and the higher presence of factor such as IL 1*β* capable of influencing Wnt pathway can potentially change the clinical outcome of OA patients and should be investigated. We strongly encourage further clinical studies associated with a quantitative analysis of the different autologous therapies used in relationship with the WNT/*β*-catenin pathway.

## 4. Discussion

In recent years, there has been a growing interest in trying to identify molecular patterns to target as a therapeutic strategy for OA, which represents the most common diseases of musculoskeletal system, involving millions of patients worldwide [51]. In line with the progress made in other fields of medicine, identifying novel pathways has led to the understanding that OA treatment should be multimodal in order to maximize the clinical outcome.

In the present review, we described one of the molecular pathways that has recently come into light for its role in OA development: the Wnt pathway. Wnt family of proteins plays a central role in several signalling cascades that are ultimately involved in increasing the inflammation in the articular environment and stimulating the release of catabolic molecules such as metalloproteinases, which are responsible for cartilage degradation, thus feeding a vicious circle between inflammation and progressive degeneration, which involves all the articular tissues [5, 6, 30, 47]. Interesting in vitro findings have shown that the Wnt pathway is significantly involved in type II collagen degradation and chondrocyte apoptosis, thus justifying the interest in deepening our knowledge of this family of proteins [33–35]. The involvement of Wnt pathway in the pathogenesis of OA has been investigated and described in many animal models, as shown by the present review: it may be argued that species-specific patterns exist that could limit the generalization of results and their application in the human setting but, despite this limitation, the search for strategies to modulate this pathway seems justified and the principal aim is to develop new pharmacological strategies that could stop OA progression at a molecular level. The most common conservative management of OA is by intra-articular injection: in recent times, traditional products such as corticosteroids and hyaluronic acid have been flanked by novel therapies based on immunomodulatory molecules and also on “autologous” derivatives such as PRP and MSCs that have the potential advantage of minimizing side effects due to their autologous nature [52]. Although limited evidence suggests that these biologic products could exert a modulation on the Wnt pathway [3, 45, 46, 53], further studies are needed to understand which mechanisms are involved in Wnt-pathway modulation and how to take full advantage of PRP and MSCs concentrate in the control of OA progression. These powerful biologic products can be regarded as a reservoir of many bioactive molecules: understanding their role and the pathways they can influence will open the possibility of manipulating these products to obtain “autologous” drugs with specific biologic actions within the joint.

There is also the opportunity for new synthetic “on-the-shelf” drugs to be developed, to influence and modulate these specific pathways. In the present review, we described some endogenous inhibitors of Wnt-pathway that could be exploited to this purpose in the near future.

Beyond the aspects related to OA therapy, the awareness on the different molecular pathways involved in OA onset and progression could stimulate further effort in the field of “early diagnosis” and “stratification” of patients affected by OA. At present, clinicians lack any reliable biomarker to identify OA in its early stages and even the definition of OA is too generic, since under this name we include very different pathological entities, ranging from posttraumatic conditions to overload-based OA (i.e., following meniscectomy) to inflammatory-based disease [54]. Each of these entities has peculiar pathogenetic features and, therefore, should be addressed differently. Therefore, the present review shows that molecular targets are among the frontiers of future therapeutic strategies in the treatment of OA: deeper insights into this field will hopefully lead to developing products, as both “intelligent” drugs and autologous derivatives, that could better modulate specific signalling pathways responsible for the progression of OA. Beyond this, better knowledge of the many molecular cascades involved in inflammatory OA will help in the future basic researchers and clinicians to stratify patients according to the molecular features of their OA, thus offering the possibility of selecting a “personalized” therapeutic approach to their disease [55].

## Figures and Tables

**Table 1:** In vitro animal studies included.

**Reference**	**Author**	**Subjects**	**Pathway involved**	**Results**
**[3]**	*J. Wu et al. (2018)*	New Zealand white rabbit chondrocytes	PRP and Wnt/ *β*-catenin	A lower expression level of *WNT1* and *β-catenin* than in another group treated with IL-1*β* was observed

**[4]**	*C.Lietman et al. (2018)*	OA-induced murine model synovial fibroblasts and chondrocytes	XAV-939	After it was injected inside the joint, a reduction of the cartilage degradation and synovial inflammation was observed

**[6]**	*L. Lodewyckx et al. (2012)*	Cell culture (ATDC5) and mouse ribcage and tibial plateau chondrocytes from *Frzb*^−/−^ and wild type mices	Wnt/*β*-catenin	Overexpression of *FRZB *coupled with an up-regulation of *aggrecan *and *Col2a1 *and down-regulation of *Col3a1 *and *Col5a1. FRZB is in strict relationship to Wnt pathway*

**[7]**	*T. Yuasa et al. (2008)*	New Zealand rabbit articular chondrocytes and guinea pig knee chondrocytes	Wnt/*β*-catenin	Wnt/beta-catenin signalling is a powerful stimulator of chondrocyte matrix catabolic action and may be part of mechanisms leading to excessive remodelling and degradation of cartilage matrix in age-associated joint pathologies

**[8]**	*M. Zhu et al. (2009)*	Adult *Col2a1-CreER*^*T2*^ transgenic mice chondrocytes	*β*-catenin	Activation of beta-catenin signalling in articular chondrocytes in adult mice leads to the premature chondrocyte differentiation and the development of an OA-like phenotype.

**[10]**	*Y. Tamamura et al. (2005)*	transgenic mice expressing a fusion mutant protein of *β*-catenin and LEF (CA-LEF) in nascent chondrocytes	*δ*-*β*-Catenin	Increase of proteins involved in cartilage matrix degradation (*MMP-13*, *ADAMTS-4*,* ADAMTS-5 *and* RUNX-2*)

**[11]**	*Shi et al. (2016)*	Sprague-Dawley rats (n=24) articular chondrocytes	WNT5A	Silencing *WNT5A* mRNA prevented degradation of *COL2*

**[15]**	*Wang D. et al (2018)*	100 BALB/c transgenic mice osteoclasts	PRP/RANKL/Wnt	The study indicated that PRP inhibits osteoclast differentiation through activation of the Wnt pathway and inhibiting RANKL

**Table 2:** In vitro human studies included.

**Reference**	**Author**	**Subjects**	**Pathway involved**	**Results**
**[5]**	*J. Wu et al. (2017)*	Human chondrocytes from OA (n=57) and healthy subjects (n=6) knees	SOST/Wnt	*SOST* may not have beneficial effects on chondrocytes affected by OA

**[9]**	*F. Dell'Accio et al. (2008)*	human articular chondrocytes (explants 24 hours after mechanical injury) from 8 patients	Wnt family	A systematic analysis of the Wnt signalling pathway revealed up-regulation of Wnt-16, down-regulation of FRZB, up-regulation of Wnt target genes, and nuclear localization of beta-catenin in injured cartilage

**[13]**	*S.-G. Gao et al. (2016)*	40 patients with various stages of primary OA cartilage and subchondral bone from tibial plateau	WIF-1	Patients with disease had significantly decreased *WIF-1* levels. Thus,* WIF-1* levels were negatively correlated with the severity of the disease

**Table 3:** In vitro both human and animal studies included.

**Reference**	**Author**	**Subjects**	**Pathway involved**	**Results**
**[12]**	*L. Chen et al. (2016)*	C57BL/6 transgenic mice and OA human (n=10) articular chondrocytes	EZH2	*EZH2* level was found significantly increased. Pharmacological inhibition of *EZH2* silenced *β*-catenin signalling pathway and delayed OA progression in mice

**[14]**	*H. Oh et al. (2012)*	Human and mouse OA model chondrocytes	DKK-1/Wnt	Overexpressing *Dkk-1* by intra-articular injection significantly reduced progression of OA in mice induced with DMM thanks to the inhibition of Wnt-mediated expression of catabolic factors

**[16]**	*Deshmukh V. et al. (2018)*	Cell culture of bone-marrow-derived human mesenchymal stem cells (hMSCs) and in vivo studies in a rodent acute cruciate ligament tear and partial medial meniscectomy (ACLT + pMMx) OA model	SM04690	SM04690 induced hMSC differentiation into mature, functional chondrocytes and decreased cartilage catabolic marker levels compared to vehicle. A single SM04690 intra-articular (IA) injection was effective in a rodent OA model

## Data Availability

All the data will be available upon motivated request to the corresponding author of the present paper.
